# The effect of warm and humidified gas insufflation in gynecological laparoscopy on maintenance of body temperature: a prospective randomized controlled multi-arm trial

**DOI:** 10.1007/s00404-022-06499-z

**Published:** 2022-03-14

**Authors:** Julia Wittenborn, Deborah Mathei, Julia van Waesberghe, Felix Zeppernick, Magdalena Zeppernick, Svetlana Tchaikovski, Ana Kowark, Markus Breuer, András Keszei, Elmar Stickeler, Norbert Zoremba, Rolf Rossaint, Christian Bruells, Ivo Meinhold-Heerlein

**Affiliations:** 1 Department of Gynecology and Obstetrics, University Hospital of the RWTH Aachen, Pauwelsstrasse 30, 52074 Aachen, Germany; 2 Department of Anesthesiology, University Hospital of the RWTH Aachen, Pauwelsstrasse 30, 52074 Aachen, Germany; 3 Department of Medical Statistics, University Hospital of the RWTH Aachen, Pauwelsstrasse 30, 52074 Aachen, Germany; 4 Department of Gynecology and Obstetrics, University Hospital of Gießen and Marburg, Justus-Liebig University Gießen, Klinikstr. 33, 35392 Giessen, Germany; 5 Department of Anesthesiology and Intensive Care, St Elisabeth Hospital, Stadtring Kattenstroth 130, 33332 Gütersloh, Germany

**Keywords:** Warm management, Body temperature, Hypothermia, Warm humidified CO_2_

## Abstract

**Background:**

Hypothermia is defined as a decrease in body core temperature to below 36 °C. If intraoperative heat-preserving measures are omitted, a patient’s temperature will fall by 1 – 2 °C. Even mild forms of intraoperative hypothermia can lead to a marked increase in morbidity and mortality. Using warm and humidified gas insufflation in laparoscopy may help in the maintenance of intraoperative body temperature.

**Methods:**

In this prospective randomized controlled study, we investigated effects of temperature and humidity of the insufflation gas on intra- and postoperative temperature management. 150 patients undergoing gynecologic laparoscopic surgery were randomly assigned to either insufflation with non-warmed, non-humidified CO_2_ with forced air warming blanket (AIR), humidified warm gas without forced air warming blanket (HUMI) or humidified warm gas combined with forced air warming blanket (HUMI+). We hypothesized that the use of warmed laparoscopic gas would have benefits in the maintenance of body temperature and reduce the occurrence of hypothermia.

**Results:**

The use of warm and humidified gas insufflation alone led to more hypothermia episodes with longer duration and longer recovery times as well as significantly lower core body temperature compared to the other two groups. In the comparison of the AIR group and HUMI + group, HUMI + patients had a significantly higher body temperature at arrival at the PACU (Post Anaesthesia Care Unit), had the least occurrence of hypothermia and suffered from less shivering.

**Conclusion:**

The use of warm and humidified gas insufflation alone does not sufficiently warm the patients. The optimal temperature management is achieved in the combination of external forced air warming and insufflation of warm and humidified laparoscopy gas.

**Supplementary Information:**

The online version contains supplementary material available at 10.1007/s00404-022-06499-z.

## Introduction

Intraoperative hypothermia is defined as a decrease in core body temperature to below 36 °C [1]. Without the use of heat-preserving measures, the temperature of most patients is reduced by 1–2 °C during an operation as their intrinsic thermoregulation switches off [2]. Due to the effect of general anesthesia, the naked body is no longer able to counteract the reduction in temperature by shivering or vasoconstriction [2]. In addition, the room temperature is often low, and cooling takes place across wound surfaces. Skin incisions for laparoscopy are much smaller than those of open surgical procedures, but the entire internal abdominal body surface interacts with the cold and dry insufflated gas (CO_2_) [3]. Therefore, the temperature drop during laparoscopic procedures does not differ essentially from that seen during open surgery. Even mild forms of intraoperative hypothermia can lead to a marked increase in morbidity and mortality [4, 5]. The negative consequences of intraoperative hypothermia have been well researched and include disturbances of blood clotting with increased blood loss as well as increased rate of transfusions, myocardial dysfunction, arrhythmia and hypokalemia [5]. In addition, delayed wound healing and wound infections occur more often, with prolonged hospitalization as a result [4–6].

CO_2_ at room temperature is usually employed in laparoscopic surgery for insufflation of the pneumoperitoneum; at room temperature it is relatively cold and dry compared with body temperature. This factor is often overlooked when intraoperative hypothermia occurs. Evidence from previous studies show, however, that the risk of hypothermia can be reduced by the use of warmed and humidified insufflation gas [7–13]. The question is especially relevant in gynecology as women in particular tend towards intraoperative hypothermia [14].

Here, we present the results of our prospective, randomized, controlled monocentric trial [A prospective, randomized, controlled, double-blinded study investigating intraoperative temperature and postoperative pain course following gynecological laparoscopy—TePaLa (Temperature and Pain in Laparoscopy)].The part concerning postoperative pain has been published before [15]. This article describes the parts of the TePaLa trial concerning the patient’s temperature during laparoscopy. We hypothesized that the use of warmed laparoscopic gas would have benefits in maintaining body temperature and in reducing the occurrence of hypothermia.

## Materials and methods

### Trial design

The study was designed as a monocentric, prospective, randomized, double-blinded controlled trial with three parallel intervention arms. It was conducted at the Department of Gynecology and Obstetrics and the Department of Anesthesiology, University Hospital Aachen, Germany between July 2016 and September 2018. Before trial commencement the study design was changed into a single-blinded trial, because the surgeon and the study staff could not be effectively blinded with respect to used devices during the laparoscopic procedure. All patients and ward staff were not aware of the used method during laparoscopy.

### Ethic committee and trial registration

The study was approved by the Ethics Committee at the RWTH Aachen University Faculty of Medicine, Germany, in August 2015. The trial is registered under the name “Temperature and Pain in Laparoscopy” (TePaLa) with ClinicalTrials.gov on May 17, 2016, trial number NCT02781194.

### Participants

150 patients with an indication to a laparoscopic gynecological surgery were randomized in 3 groups of 50 subjects each. In group 1 (AIR), a forced air warming blanket and cold and dry insufflation gas has been used during surgery. In group 2 (HUMI), insufflation has been performed by warm and humidified insufflation gas and no warming blanket was used. Group 3 (HUMI+) was treated with a combination of a forced air warming blanket and warm, humidified gas.

### Inclusion criteria

Eligible patients were female, aged between 18 and 69 years with a body mass index under 35, admitted for laparoscopic surgery with a planned duration of more than 60 min.

### Exclusion criteria

Exclusion criteria were pregnancy or no sufficient contraception, women, who are breastfeeding, alcohol or drug abuse, expected non-compliance, patients unwilling or unable to give informed consent, patients with limited ability to comply with instructions for this study, participation in another interventional study within the last 3 months, subjects who are committed to an institution and/ or penitentiary by judicial or official order and employees of the investigator cooperation companies.

The study could have been terminated due to a violation of the study protocol, the declaration of Helsinki, ICH-GCP and/or applicable regulatory requirements. Participants of the study were excluded if the patient withdrew informed consent or did not follow instructions by the study team, if the safety and well-being could not be ensured any longer, if the risks and benefits of continuing the study had been reassessed, and the risk outweighed any potential benefit, if the incidence of adverse events (AE) constituted a potential health hazard to the subjects, if perioperative temperature dropped below 35.0 °C (core body temperature), and if intraoperative loss of blood was more than 500 ml.

Following physical examination and elucidation about the study course by a senior physician, eligible patients were requested to participate in the study.

### Outcomes

The primary endpoint was the occurrence and total number of episodes of hypothermia (< 36 °C). Their duration and the time until recovery were secondary endpoints as well as the dynamic of the body temperature and its time course throughout the operation until the arrival in the post-anesthesia care unit (PACU) and the duration of stay at the PACU.

### Randomization

After written informed consent was obtained, study participants were randomized with equal allocation ratios to the three interventions using permuted block randomization (block size 6) stratified by endometriosis (Yes/No). Computer generated sequences were used. To maintain allocation concealment, the randomization sequence and the block size were concealed from the investigators and the study team until database lock, and the assignment to study participants was carried out with a web-based application maintained by the Institute of Medical Informatics, RWTH Aachen University.

### Interventions

#### Pre-surgical treatment

Patients were warmed with a duvet for one hour before the procedure in the holding area of the operation theatre. An underbody blanket for delivery of forced air warming was placed on every procedure table before patients were placed on it but was used only in groups AIR and HUMI+. This ensured allocation concealment for patients and provided a possibility of external warming up in case of lowering the core body temperature below 35 °C in the HUMI group (exclusion criterion). During transfer from the holding area to the operation room, all patients were covered with prewarmed cotton sheets. The ambient temperature of anesthetic preparation room and operation theatre was 21 °C, measured and set by the central air conditioning system. One hour prior to the induction of general anesthesia, all patients received tympanal temperature measurements every 10 min.

#### Intraoperative procedures

In case if epidural anesthesia was indicated and desired by the patient, the epidural catheter was placed according to standard operating procedure. All patients received general anesthesia as total intravenous anesthesia or low flow (< 1 l/ min) balanced anesthesia. After induction of anesthesia patients of the AIR and HUMI + group received forced air warming, administered by the 3 M Bair Hugger Warming Unit, Model 775 in combination with 3 M Bair Hugger Lithotomy Underbody Blanket, Model 585 (both 3 M company, St Paul, USA). Patients of the HUMI group were covered only with cotton sheets. An esophageal thermometer was placed immediately after induction of general anesthesia and temperatures were taken every 10 min for the entire duration of the operation. All participants underwent laparoscopic surgery in a lithotomy position, which was performed by one of four surgeons appointed by the principal investigator because of their similar surgical technique. According to randomization, capnoperitoneum was established and maintained either with cold and dry CO_2_ (21.0 °C room temperature/ 0% humidity) in the AIR group or with warm and humidified CO_2_ (depending on flow rate > 38.6 °C/ > 98%)[16] in the HUMI and HUMI + group. Insufflation gas was warmed and humidified by the F&P HumiGard Surgical Humidifier MR860 (Fisher & Paykel Healthcare Limited, Auckland, New Zealand). Heated and humidified gas was supplied by the ST310 Laparoscopic Humidification Kit (Fisher & Paykel Healthcare Limited, Auckland, New Zealand). The actively heated tube maintained the temperature and humidity of the gas until it was delivered to the patient interface (37.0 °C ± 0.8/ 100.0% ± 0.05)[17]. For CO_2_ insufflation, 26432020-1 THERMOFLATOR from KARL STORZ was used (KARL STORZ SE & Co. KG, Tuttlingen, Germany). Maximum gas pressure was set to 15 mmHg, so that except for the laparoscopic port entry procedure, the maximum intraperitoneal pressure did not exceed this limit. Fluid management contained an intravenous fluid input of minimum 4 ml/kg/h with an average aim of 500 ml/h. Intravenous fluids were administered via HL-90-DE-230 HOTLINE Blood and Fluid Warmer (Smiths Medical, Inc., Minneapolis, USA) in all groups.

For the patients transfer from the operating theatre to the PACU, patients were again covered with prewarmed cotton sheets.

#### Post-surgical data acquisition

At the time of arrival at the PACU, the tympanal temperature was measured again. In case, the measurement was below 36.5 °C, repeated measurements were performed until the patient’s body temperature reached 36.5 °C. The occurrence of postoperative shivering was noted.

### Statistical analysis

Outcome variables were described within each treatment group using standard descriptive statistics (frequency, minimum, maximum, quartiles, mean, and standard deviation). The occurrence of episodes of hypothermia (< 36 °C), their duration and the time until full recovery was analyzed by group. Body temperature data were analyzed using a linear mixed effects model [18, 19]. The model included fixed effects for the treatments, measurement occasions, stratification and randomization blocks, as well as measurement-treatment interactions. The random part included intercepts grouped by subjects. We based the inference on the treatment effect on a likelihood ratio test comparing the full model with a restricted model excluding all treatment effects. Estimated treatment effects at each measurement occasion were calculated from the model along with nominal 95% confidence intervals. Furthermore, the dynamic of the body temperature during the whole duration of the operation until the arrival in the PACU was compared between the different groups.

Analyses were conducted using R [20]. Mixed models were fitted with lme4 [21].

## Results

### Study population

A total of 208 patients with an indication to a laparoscopic gynecologic surgery were assessed for eligibility between July 2016 and September 2018. 58 subjects were not included, as they did not meet inclusion criteria, declined to participate, participated in another study or for other reasons. 150 patients, who finally were included into the study, were randomized either to the control group or to 1 of the 2 intervention groups.

One patient randomized to the control group (AIR) accidently received no warming blanket, but warm humidified insufflation gas instead of the allocated intervention with forced air warming blanket alone. One patient randomized into the HUMI group did not receive surgery, and consequently the allocated intervention, because of preoperative spontaneous rupture of the ovarian cyst that was the indication for laparoscopy. Two patients randomized into HUMI+ group did not receive the allocated intervention: in one case, because the planned surgery was not performed due to loss of indication after randomization and one patient received forced air warming blanket only instead of the allocated intervention.

In two cases, the intervention was discontinued: one patient from the AIR group, because of the intraoperative indication to a conversion of the laparoscopic procedure into laparotomy, and one patient from the HUMI group, because of the lowering of body core temperature below 35 °C during the intervention with the necessity to use additionally forced air warming blanket.

**Fig. 1:**
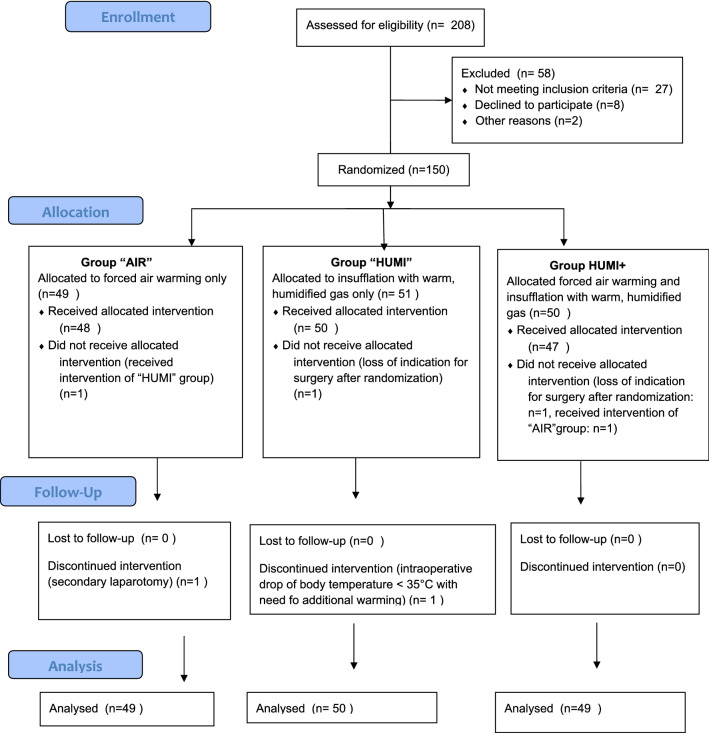
Consort flow diagram

Primary—Intention to treat—analysis was performed (Fig. 1).

#### Intraoperative variables

Table 1 shows the baseline characteristics of all patients who received surgery. Demographic data, risk factors for cardiovascular complications, patient’s medical history, intraoperative medication, fluids and insufflated CO_2_ as well as the type and length of operative procedures were recorded. No significant differences between the groups were recorded.

**Table 1:** Baseline Characteristics of the investigated patient groups (all participants who underwent surgery). This table was published before in the part describing postoperative pain [13]

	AIR group	HUMI group	HUMI + group	*P* value
	*n* = 49	*n* = 50	*n* = 49	
Age (years)	40.4 ± 14.0	36.1 ± 11.7	38.7 ± 11.6	0.51
BMI	24.7 ± 3.78	26.1 ± 4.71	23.0 ± 3.55	0.05
Smoker	16 (32.7)	18 (36.0)	7 (14.3)	
Cigarettes per day	13.5 ± 6.23	11.9 ± 6.82	11.1 ± 7.45	0.39
Smoking years	16.3 ± 14.5	16.2 ± 14.6	14.2 ± 7.6	0.79
Ex-Smoker	8 (16.3)	11 (22.0)	14 (28.6)	
Cigarettes per day	12.5 ± 6.89	16.1 ± 6.97	11.5 ± 6.17	0.53
Smoking Years	9.57 ± 7.91	11.30 ± 9.38	9.79 ± 6.89	0.96
Risk factors for CV complications				
Hypercholesterolaemia	3 (6.1)	2 (4.0)	3 (6.1)	0.82
Hypertension	9 (18.4)	5 (10.0)	4 (8.2)	0.26
Overweight	19 (38.8)	24 (48.0)	12 (24.5)	0.05
Co-Morbidities				
Diabetes	3 (6.1)	1 (2.0)	3 (6.1)	0.57
Arteriosclerosis	1 (2.0)	0	0	0.66
Asthma	7 (14.3)	3 (6.0)	2 (4.1)	0.21
Thyroid dysfunction	7 (14.3)	14 (28.0)	9 (18.4)	0.22
Previous abdominal surgery				
Laparoscopic	22 (44.9)	20 (40.0)	23 (46.9)	0.31
Abdominal	12 (24.5)	12 (24.0)	6 (12.2)	
Type of surgical procedure				
Endometriosis	19 (38.8)	24 (48.0)	20 (40.8)	0.62
Hysterectomy	12 (24.5)	14 (28.0)	18 (36.7)	0.39
Myomectomy	7 (814.3)	5 (10.0)	4 (8.2)	0.61
Cyst removal	8 (16.3)	8 (16.0)	4 (8.2)	0.41
Salpingo-oophorectomy	9 (18.4)	5 (10.0)	9 (18.4)	0.41
Other	13 (26.5)	19 (38.0)	18 (36.8)	0.42
Length of surgery (min)	169 ± 97.8	166 ± 84.6	171 ± 92.6	0.96
Amount of intraperitoneal irrigating fluids (ml)	849 ± 600	971 ± 597	976 ± 611	0.54
Length of capnoperitoneum (min)	109 ± 78.0	109 ± 77.1	116 ± 73.1	0.88
Amount of insufflated CO_2_ (l)	294 ± 338	261 ± 237	294 ± 229	0.79
Epidural catheter	11 (28.2)	16 (41.0)	12 (30.8)	0.52
Anaesthetics				
Propofol (mg/h)	419 ± 59.4 (*n* = 20)	437 ± 86.0 (*n* = 14)	387 ± 68.4 (*n* = 22)	0.1
Sevoflurane (%)	1.57 ± 0.290 (*n* = 28)	1.56 ± 0.285 (*n* = 37)	1.55 ± 0.213 (*n* = 22)	0.98
Desflurane (%)	5.00 (*n* = 1)	5.33 ± 0.808 (*n* = 3)	5.10 ± 0.316 (*n* = 5)	0.8
Intraoperative opioids				
Sufentanil (µg)	45.1 ± 15.1 (*n* = 47)	40.2 ± 13.0 (*n* = 50)	45.5 ± 18.3 (*n* = 48)	0.17
Remifentanil (µg/ h)	43 (*n* = 1)	1200 (*n* = 1)	(*n* = 0)	
Fentanil (mg)	0.5 ± 0.1 (*n* = 2)	(*n* = 0)	0.4 (*n* = 1)	
Piritramide (mg)	5.28 ± 2.28 (*n* = 29)	5.27 ± 2.33 (*n* = 32)	4.75 ± 2.15 (*n* = 23)	0.64
Intraoperative non-opioids				
Metamizole (g)	1.20 ± 0.391 (*n* = 23)	1.21 ± 0.379 (*n* = 26)	1.19 ± 0.385 (*n* = 24)	0.98
Paracetamol (g)	1 ± 0 (*n* = 8)	1 ± 0 (*n* = 1)	1 ± 0 (*n* = 4)	0.03
Ibuprofen (g)	0	0.525 ± 0.125 (*n* = 2)	0.4 ± 0 (*n* = 2)	
Intraoperative Relaxant				
Rocuroniom (mg)	49.9 ± 21.1 (*n* = 49)	53.5 ± 23.7 (*n* = 50)	51.4 ± 19.7 (*n* = 49)	0.71
Length of anaesthesia (min)	193 ± 104.0	187 ± 90.8	193 ± 94.2	0.94
Amount of infusions (ml)	1638 ± 796	1690 ± 748	1714 ± 661	0.87

#### Primary endpoint-intraoperative occurrence of hypothermia

The total number of hypothermia episodes (< 36 °C) differed significantly between the three groups (see Table 2).

**Table 2:** Total number of hypothermia episodes (< 36 °C), rate of hypothermic measurements of all temperature measurements in the respective group [%], rate of hypothermic measurements per 10 min interval

	Total number of hypothermia episodes (< 36 °C)	Rate of hypothermic measurements (%)	Rate (Events per 10 min interval)
Group AIR	45	3.8	0.036
Group HUMI	277	20.5	0.19
Group HUMI+	53	4.3	0.041

During anaesthesia and postoperatively, there were significantly more temperatures below 36 °C in the HUMI group than in the AIR or HUMI+ group. In the AIR and HUMI + group, hypothermic measurement were rare (Table 3).

**Table 3:** Hypothermia episodes over time

Period	Group	Total number of hypothermia episodes	% of all temperature measurements in the respective period
Preoperatively (1 h prior anaesthesia induction)	AIR	0	0
	HUMI	0	0
	HUMI+	0	0
During anaesthesia (entire period of anaesthesia)	AIR	42	5.1
	HUMI	209	25
	HUMI+	49	5.8
Postoperatively (arrival at PACU until temperature > 36.5 °C)	AIR	3	3.6
	HUMI	68	36.2
	HUMI+	4	4.5

#### Duration of hypothermia

**Fig. 2:**
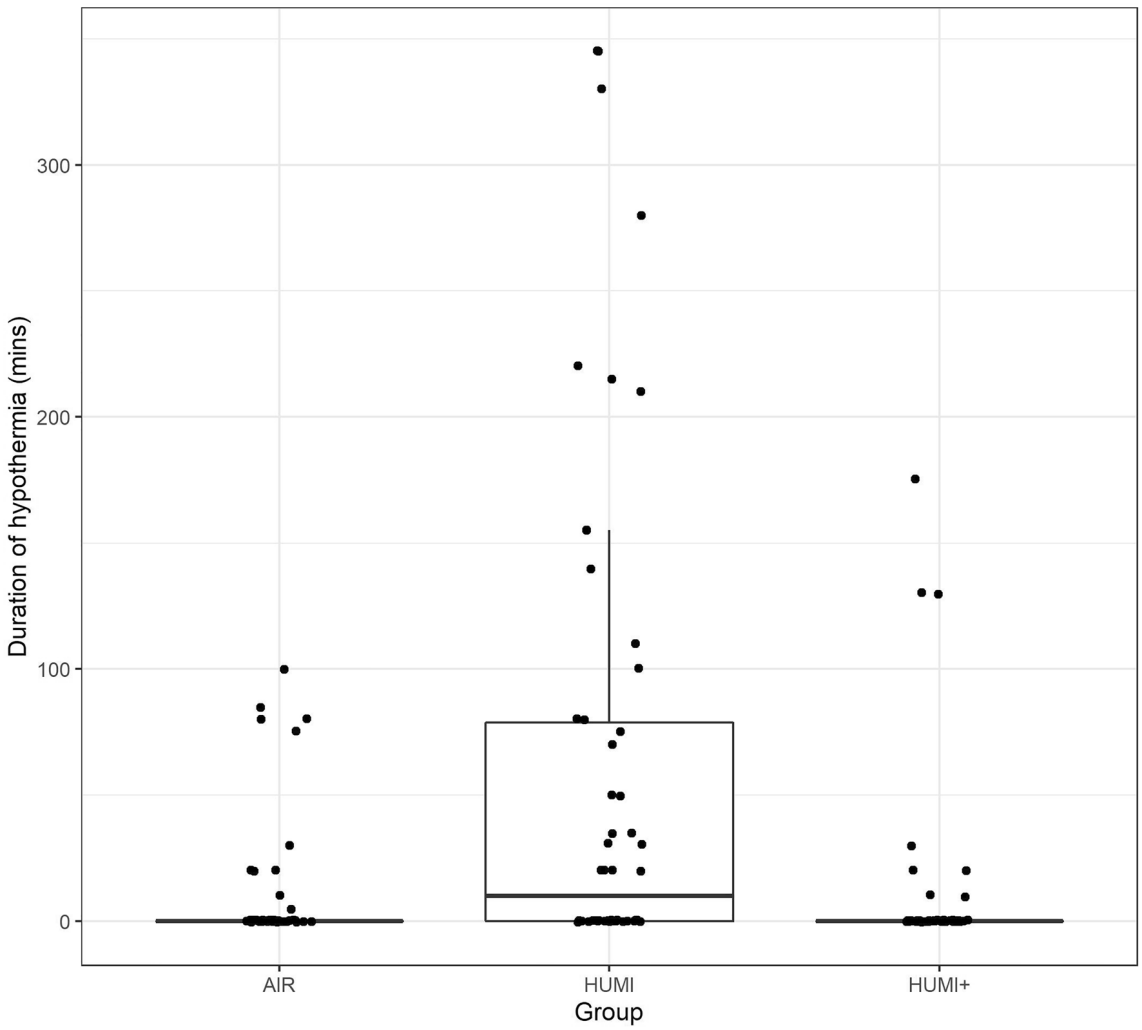
Duration of hypothermia: Boxplots of duration of hypothermia in the TePaLa study by intervention groups. Dots represent jittered data points

The duration of hypothermia was significantly longer in the HUMI group than in the other two groups. In the AIR group, 22% of the patients suffered from hypothermia; in the HUMI+ group, it was 16% of all patients. In comparison, in the HUMI group, every second patient had at least one hypothermic episode (see Table 4, Fig. 2).

**Table 4:** Duration of hypothermia episodes, proportion of hypothermic patients

Gruppe	Cumulative duration of hypothermia episodes (min)	Patients with at least one measurement < 36°
AIR	10.7	11 (22%)
HUMI	61.3	25 (50%)
HUMI+	10.7	8 (16%)

#### Recovery time

**Fig. 3:**
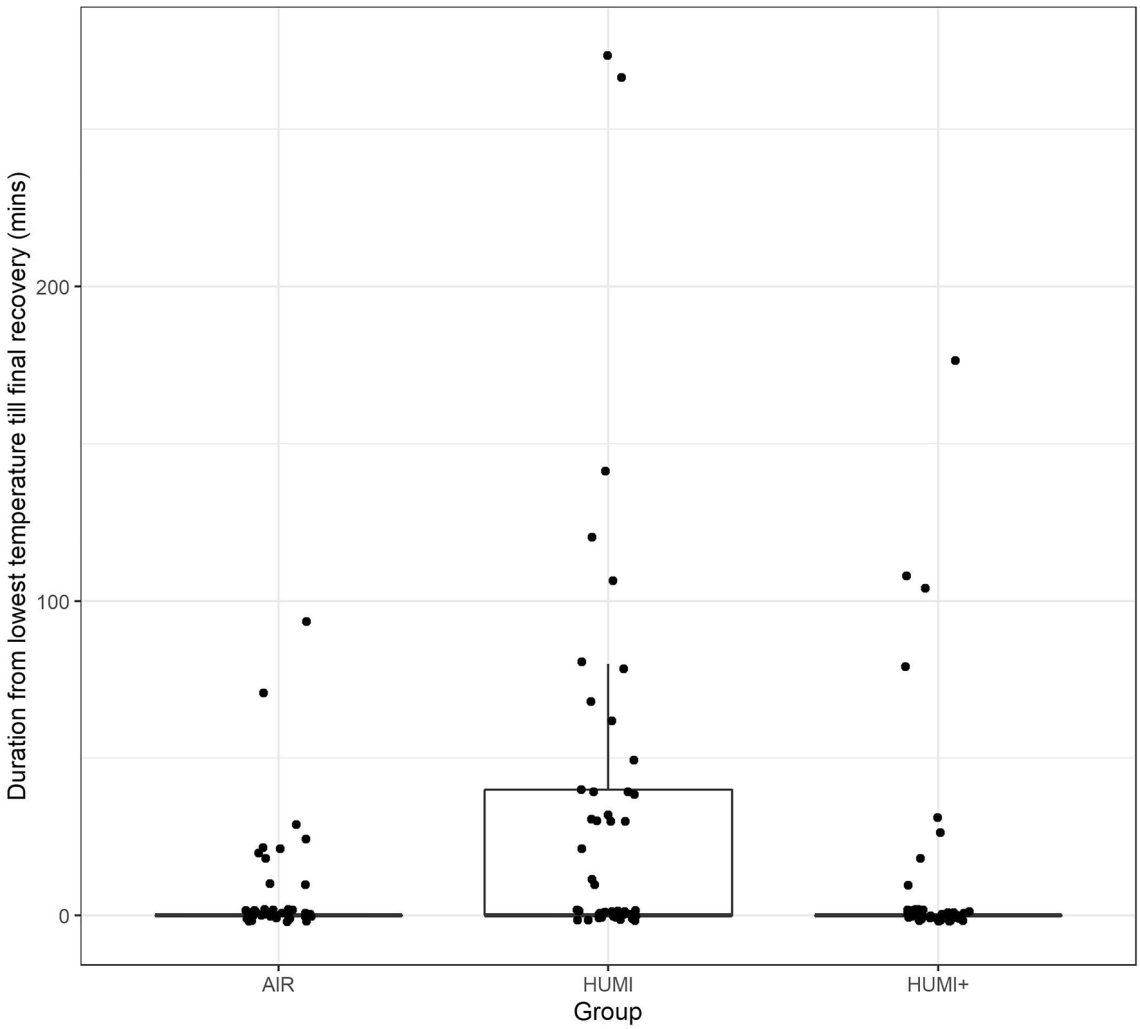
Recovery time: Boxplots of time duration from the lowest measured temperature < 36 °C to final temperature recovery, defined as earliest temperature measure >  = 36 °C after hypothermia that was not followed by any subsequent measurement < 36 °C, plotted by intervention groups. Dots represent jittered data points

The time from the lowest measured temperature until the first measurement of a temperature > 36 °C was defined as recovery time. This time interval also differed significantly between the three groups (see Table 5, Fig. 3). In group 2 (HUMI), three patients did not recover from hypothermia within the study, in the AIR group, it was one, whereas all patients in group 3 (HUMI+) showed recovery within study time.

**Table 5:** Recovery time, number of patients without intraoperative recovery

Group	Recovery time (min)	Number of patients without intraoperative recovery
AIR	6.7	1
HUMI	33.9	3
HUMI+	11.3	0

#### Body temperature

**Fig. 4:**
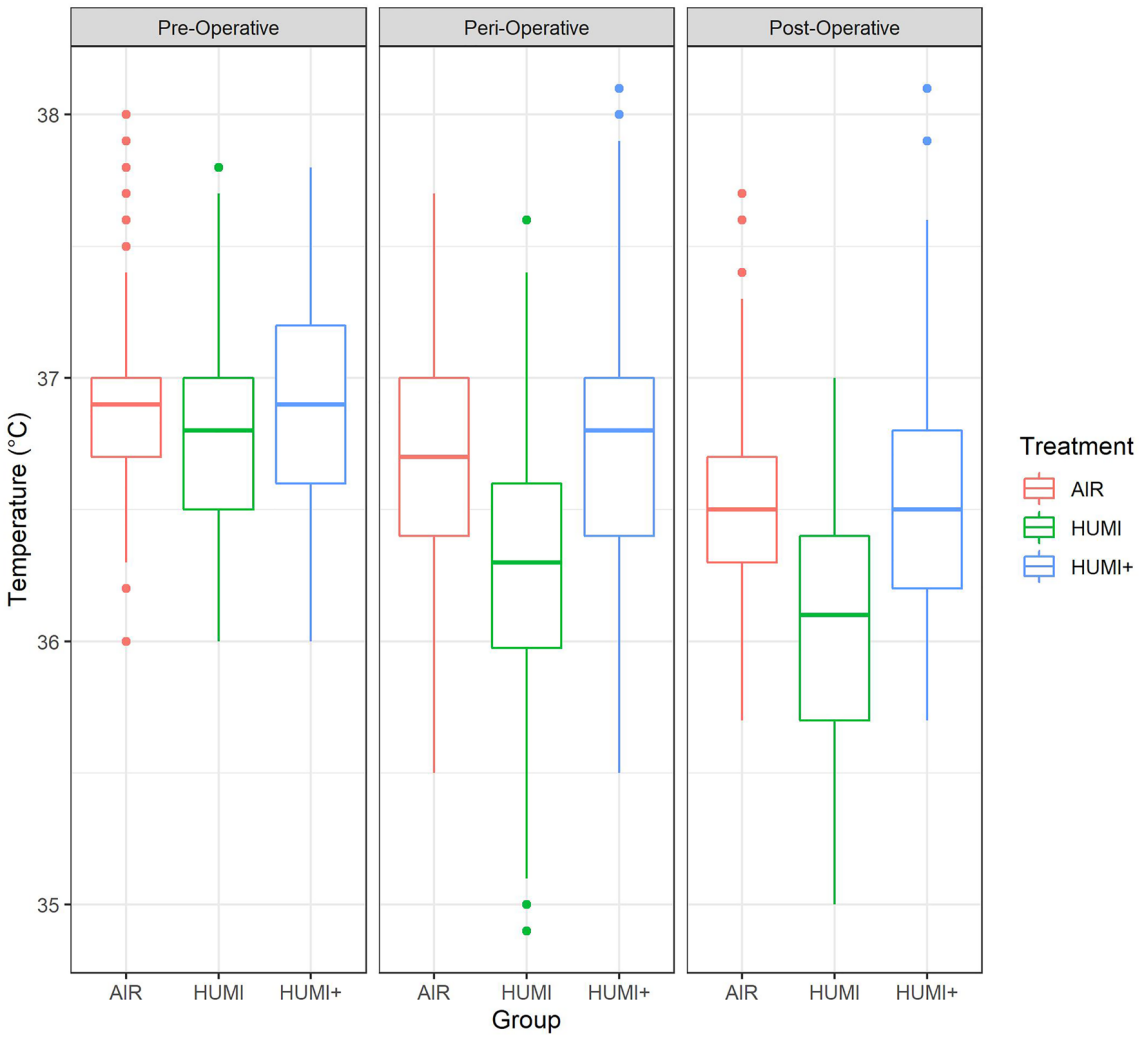
Boxplot of pre-, peri- and postoperative temperature measurements by intervention groups

Median preoperative body temperature measurements did not differ significantly. Perioperatively, the median temperature was highest in group 3 (HUMI+); in group 2 (HUMI), the temperature measurements dropped significantly compared to group 1 (AIR) and 3 (HUMI+) (Fig. 4).

#### Differences in temperature by groups

**Fig. 5:**
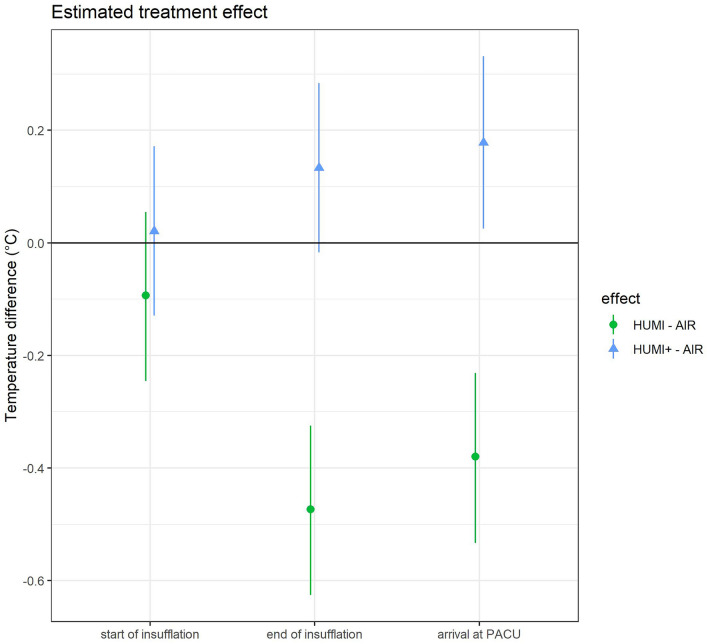
Estimated treatment effects on temperature (relative to intervention group AIR). Line ranges are pointwise 95% confidence intervals. Treatment effects are estimated using a linear model with timepoints (1,2,3,4), treatment (1,2,3) and time point interactions (no treatment main effect), stratification (1,2), and blocks (1 to 6) as fixed effects and random intercepts grouped by subjects

To evaluate the effect of Humigard® on body temperature, we assessed the temperature difference at certain time points between HUMI and HUMI + group in comparison to the AIR group. The results are depicted in Fig. 5, Table 6.

**Table 6:** Temperature differences at time points 2, 3 und 4 in comparison to group 1

Time point	Compared groups	Effect	*p* value
2 = start of insufflation	HUMI vs AIR	− 0.093	0.213
	HUMI + vs AIR	0.021	0.788
3 = end of insufflation	HUMI vs AIR	− 0.473	< 0.001
	HUMI + vs AIR	0.133	0.087
4 = arrival at PACU	HUMI vs AIR	− 0.38	< 0.001
	HUMI + vs AIR	0.178	0.025

Treatment 2–1 (green circle) describes the estimated difference between group 2 (HUMI) versus group 1 (AIR) with a 95% confidence interval (*p* value < 0.05). The measured temperatures in the HUMI group are significantly lower compared to the AIR group at time point 3 and 4.

Treatment 3–1 (blue/triangle) describes the difference between group 3 (HUMI+) versus group 1 (AIR). Temperatures were higher in HUMI+ group at all three time points, the result was statistically different at time point 4 (Table 6).

To validate this data, we analysed the presence of potential confounders. As potential confounders high BMI (kg/m^2^), quantity of warmed infusions (in ml/h) and duration of gas insufflation (in min.) were investigated. None of the above-mentioned potential confounders had an influence on the results of the calculation.

#### Temperature trends by groups

**Fig. 6:**
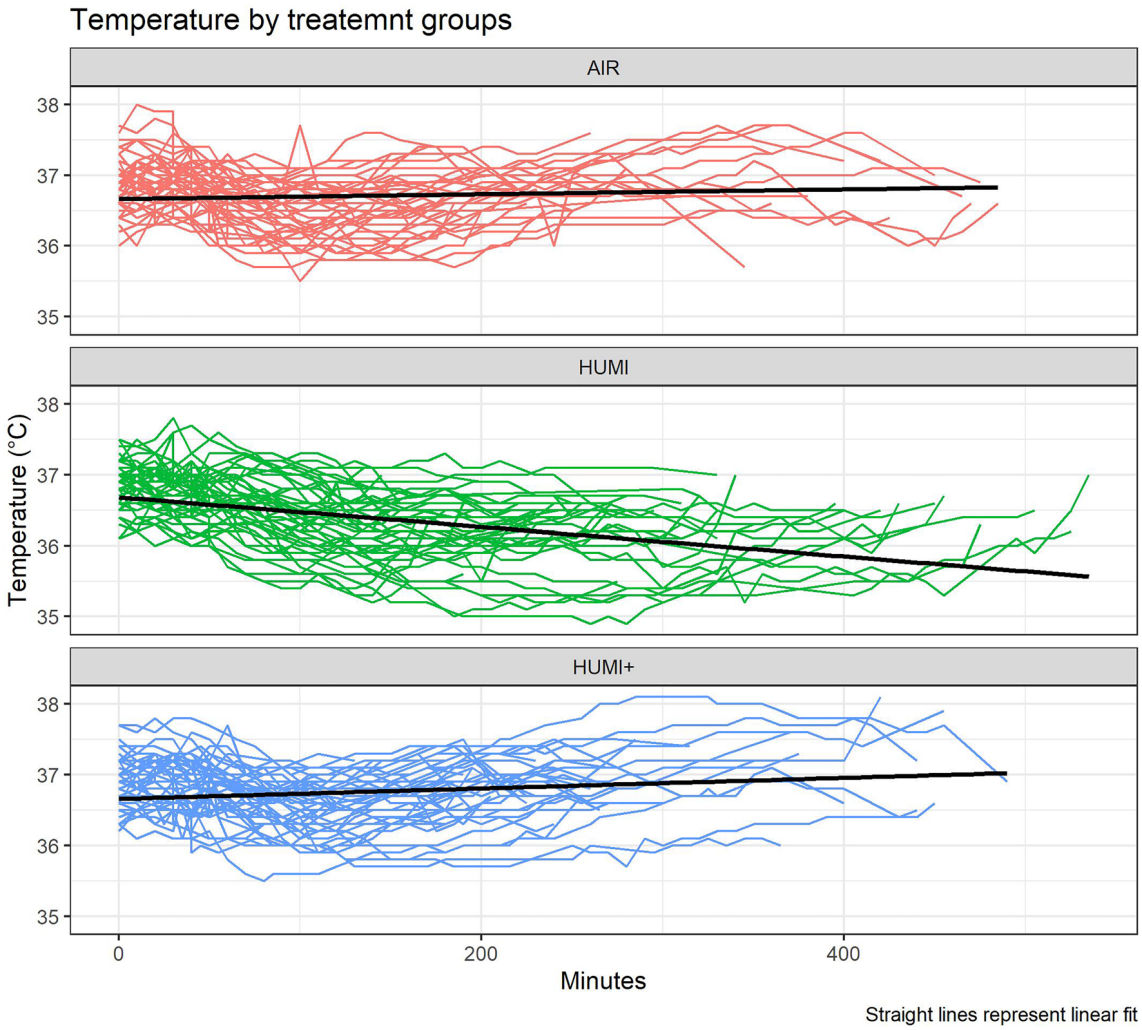
Temperature trends after averaging all temperature data within the different groups. The straight fat line depicts the slope

All temperature data of the different groups were included into a temperature trend estimation in terms of a positive or negative Slope (°C/min) (see Fig. 6).

A positive trend was detected in the AIR group (slope: 0.001637) and in the HUMI+ group (slope: 0.002218). HUMI+ group showed a higher slope than the AIR group. In the HUMI group, the trend was negative (slope: − 0.000453) (Table 7).

**Table 7:** Slopes of all temperature data of the different groups

Group	Slope (°C/min)
AIR	+ 0.001637
HUMI	− 0.000453
HUMI+	+ 0.002218

#### Length of PACU stay

As a secondary endpoint, we analysed the length of PACU Stay. Usually, patients have to be normothermic, non-shivering and without nausea, vomiting or pain as well as cardiopulmonary stable before they can be transferred to the wards [22].

**Fig. 7:**
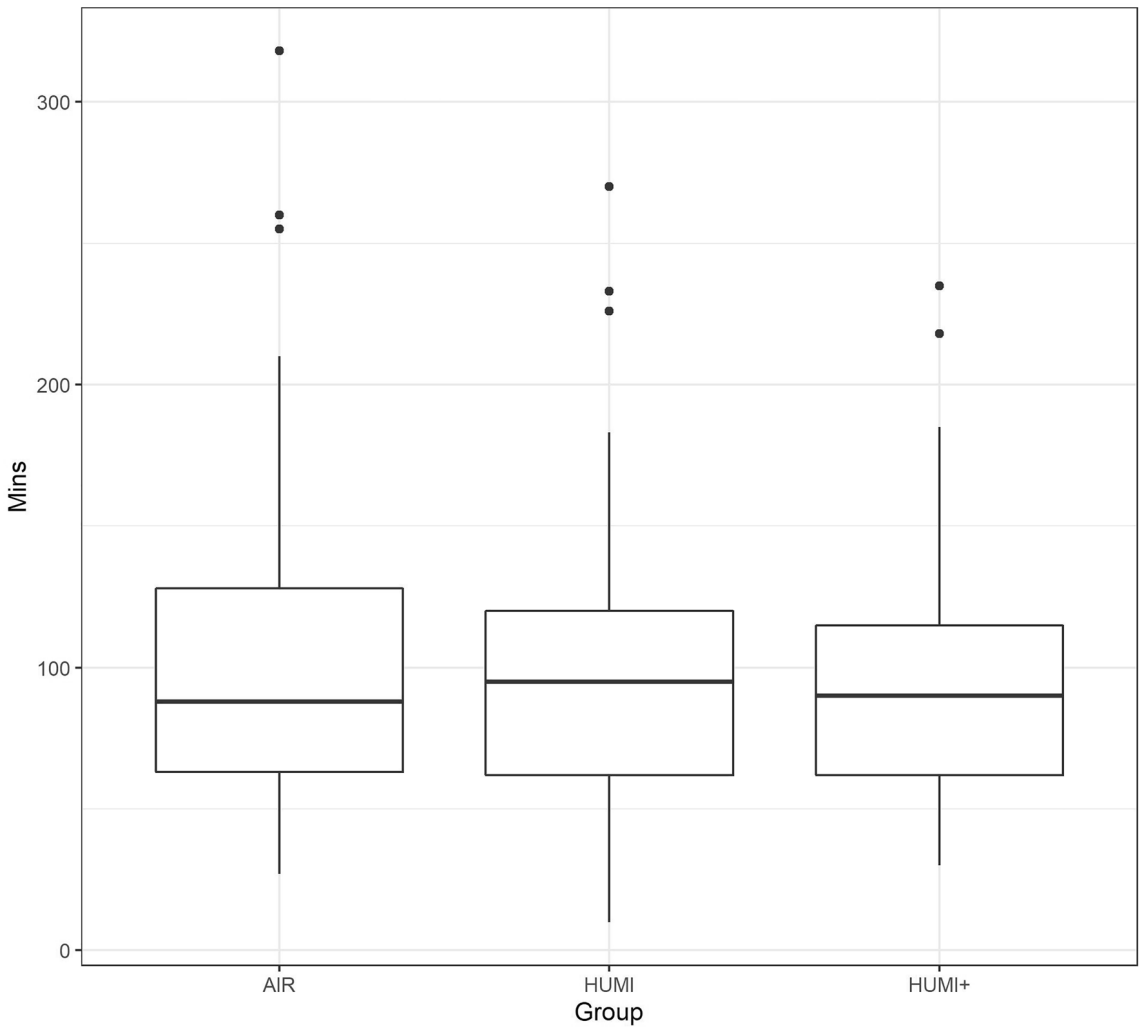
Boxplot: Duration of PACU stay (in min) by groups

The average duration of the PACU stay was 104 min. in the AIR group, 98 min. in the HUMI group and 96 min. in the HUMI+ group (see Fig. 7).

Patients who were intraoperatively warmed with Humigard® (HUMI) or Humigard® and BairHugger® (HUMI+) had shorter stays in PACU than patients warmed with BairHugger® alone (AIR).

#### Shivering

In the HUMI group, 34% of the patients suffered from shivering, in the HUMI+ group only 6%. Shivering occurred in 20% of patients in the AIR group (see Table 8).

**Table 8:** Occurrence of shivering

Group	Shivering	No shivering
AIR	10 (20%)	39 (80%)
HUMI	17 (34%)	33 (66%)
HUMI+	3 (6%)	46 (94%)

#### Adverse events

A total of 109 (74%) study objects experienced adverse events during study intervention. All adverse events were classified as mild and they were not, or highly unlikely related to the study (see Supplement, Table 9).

## Discussion

In the present study, we investigated the effect of three different intraoperative temperature management regimen using either standard conditions with heating blanket with forced air warming and dry insufflation gas at room temperature (AIR), no heating blanket, but warm and humidified insufflation gas (HUMI) or both, heating blanket combined with forced air warming and warm and humidified insufflation gas (HUMI+). Although it was clearly shown that warm and humidified insufflation gas alone does not sufficiently warm the patients, we could work out that adding warm and humidified insufflation gas to the standard conditions with a forced air warming blanked has advantages for the patients regarding intra- and postoperative maintenance of body temperature. Both has been unknown from our knowledge.

We identified two recent meta-analyses and a Cochrane review addressing the topic of warmed, humidified carbon dioxide insufflation in laparoscopic surgery. Balayssac et al. found significantly higher core temperatures of patients when using warmed and humidified gas compared to standard, but were not able to show positive effects in postoperative core temperatures [23]. In the latest Cochrane analysis, a slightly higher body core temperature was confirmed. However, the benefit disappeared when the analysis only included trials with a known low risk of bias [24]. Dean et al. found a significant difference in mean core temperature change between the warmed, humidified group and the cold, dry group, with an effect size of + 0.3 °C. All three analyses underline the great heterogeneity between the studies.

We present a monocentric study, which, in our opinion has certain advantages: The whole perioperative setup from taking the patients to the operating theatre until release from the PACU followed a strict protocol, including a constant and comparable operating room temperature and a constant surgical team. Temperature was measured in the esophagus, which is the most reliable and valid measurement of the body temperature [1, 25].

When looking at our data, warm and humidified gas insufflation alone has a clear disadvantage in keeping patients warm in contrast to the combination of both heating blanket with forced air warming and warm and humidified insufflation gas or heating blanket with forced air warming alone. Of all the studies looking at warm, humidified gas insufflation only three mentioned the use of external warming. In all three studies, it was used on the control group and the intervention group [12, 26, 27]. Our study is the first to investigate and compare warm, humidified gas insufflation alone against external warming alone. The results show a very clear disadvantage for warm and humidified gas insufflation alone regarding the intra-and postoperative body temperature, the occurrence and the duration of hypothermia and the recovery from hypothermia.

A possible reason for this might be the fact that in the operating theatre it often comes to waiting time (e. g. during patient preparation, waiting for the operative team or for the system to be installed or connected), during which the forced air warming blanked is already in action, whereas the humidified gas insufflation is not. To reduce this “gap” in the patient warming process, Humigard should not be used alone [28–30].

### Comparison of AIR and HUMI+ group

Looking at potential differences between the forced air warming alone group and the group combining forced air warming with insufflation of humidified, warm gas (group AIR vs HUMI+) slight differences can be distinguished.

Although temperature levels were very similar in both groups (AIR and HUMI+), temperature levels in the HUMI+ group were higher at all regarded time points and statistically significantly higher at the arrival in the PACU. This positive finding is underlined by the fact that the rate of patients with at least one measurement of body temperature below 36 °C is lowest in group 3. In contrast to the other two groups, patients with the combined warming approach (HUMI+) had a 100% recovery rate from hypothermia. This result is in contrast to Nguyen et al. who concluded that the addition of heated and humidified carbon dioxide gas is neglectable [26]. This may be due to the fact that their sample size with ten patients per group was too small. The findings confirm our previously published results of a retrospective case–control analysis underlining the positive effect of the addition of warm and humidified gas insufflation [13].

Our data suggest that the treatment effect of the additional humidified and warm gas insufflation is highest at the end of the operation (see Figs. 5 and 6). This finding is in accordance with the meta-analysis of Dean et al. who showed a significant difference of body temperature in studies applying forced air warming additionally to humidified, warmed gas insufflation only in patients with operating time > 80 min [29]. The explanation lies within the pathophysiology of anaesthesia induction: The inhibition of vasoconstriction has the effect of core-to-peripheral redistribution of body heat, decreasing core temperature. This can best be antagonized via prewarming and external forced air warming [13, 28, 30]. After this redistribution, which causes the greatest heat loss, the effect of warm, humidified gas insufflation can kick in.

### Postoperative body temperature

At the time of arrival in the PACU, we were able to show a treatment effect by the addition of humidified, warm gas insufflation. Other randomized controlled double-blinded studies that could not confirm a potential benefit of adding warm, humidified gas insufflation stopped taking temperature measurements at the end of surgery and let the temperature of intravenous fluid given intraoperatively up to the discretion of the anesthetic team [27]. We hypothesize that this conclusion could have been confounded by the uncontrolled application of warm intravenous fluids in former studies. To exclude this bias temperature of fluids was controlled in our study. Additionally, we observed the patient’s body temperature for a longer period of time. In our opinion, this is quite important, as many of the adverse events caused by hypothermia occur in the postoperative period as well (e.g. arrythmia, myocardial dysfunction, delayed wound healing) [1, 31].

Postoperatively, even small deviations in body temperature can result in shivering and impairment of well-being and in our opinion should therefore be attentively considered [1, 31, 32].

### Shivering

Interestingly, we found the lowest occurrence of shivering in the HUMI + group. It affected only 6% of the patients in this group. In the other groups, the rates were much higher: 34% in the Humi group and 20% in the AIR group. Therefore, in comparison to the AIR group, the addition of warmed and humidified insufflation gas resulted in 28% less shivering (Table 8). This is a fundamental difference that should be kept in mind as shivering is one of the leading causes of discomfort for postsurgical patients. It is usually triggered by hypothermia and causes involuntary contraction of skeletal muscle. It serves exclusively to produce heat (thermogenesis), with little of the energy expended as physical work and hampers patients’ conditions especially with reduced cardiac function unable to increase oxygen delivery.

### Confounders

Adipose patients with a BMI > 35 were excluded from the study, because it can be assumed that due to the isolating properties of adipose tissue, hypothermia occurs later in these patients [33]. To rule out all possible confounders we corrected our results by BMI (kg/m^2^), amount of warmed infusions and insufflation time. It did not change significance levels of our results.

## Limitations and strength

Our study has several strengths and limitations that need to be addressed. First, the study design had a clear randomization, single blinding, a pre-defined surgical team and a reliable and valid measurement of patients’ body temperature in the esophagus. All factors known to interfere with patients’ body temperature were controlled and valid results could be achieved.

It can be argued that a potential weakness of the study was to be performed mono- and not multicentric, although we believe that this limitation might add to the validity of our data as confounding factors could be reduced given the constant perioperative setup as discussed above.

Other presumed effects of warm humidified gas insufflation like the reduction of postoperative adhesions were not investigated in this study. The trial design was tailored to evaluate the effect on body temperature and therefore did not include second-look laparoscopies which would have been necessary to answer the question of adhesion formation. The effect on postoperative pain was also investigated in this study and published before [15].

It is of interest whether the results of this monocentric study can be extrapolated on other patient populations (e.g. urologic, etc.) or reproduced in other hospital settings.

The adverse events we recorded were overall mild and not related to the study-nevertheless unlikely simply by the use of differently warm and humid insufflation gas.

## Conclusion

The application of warm, humidified gas in laparoscopy alone is not suitable for adequate perioperative heat-preserving and has clear disadvantages compared to external warming. Combining external warming and the insufflation of warm and humidified gas resulted in the best temperature management for the patients and should, therefore, be considered for optimizing perioperative outcomes.

## Supplementary Information

Below is the link to the electronic supplementary material.

Supplementary file1 (DOCX 13 kb)
