# Supplementary material for: The effect of warm and humidified gas insufflation in gynecological laparoscopy on maintenance of body temperature: a prospective randomized controlled multi-arm trial
Source: Arch Gynecol Obstet. 2022 Mar 14;306(3):753–67. doi: 10.1007/s00404-022-06499-z (PMC9411231; doi:10.1007/s00404-022-06499-z)
Supplement: Supplementary file 1 — Supplementary file1 (DOCX 13 kb) [file 404_2022_6499_MOESM1_ESM.docx]

Table 9: list of adverse events in interventional and control groups

| Time point | AIR | HUMI | HUMI+ |
| --- | --- | --- | --- |
| AV-Block III° | 0 | 0 | 1 |
| Bradycardia | 30 | 31 | 33 |
| Bradycardia and hypotension | 0 | 1 | 0 |
| Bradycardia and hypthermia | 0 | 1 | 0 |
| Tachycardia | 1 | 2 | 0 |
| Hypertension | 1 | 0 | 1 |
| Hypotension | 1 | 4 | 2 |
